# Defining Genetic Ancestry: Implications for Nurses

**DOI:** 10.1177/10998004251374146

**Published:** 2025-08-27

**Authors:** Alexis Jimenez, Karla Lindquist, Kayla D. Longoria, Benjamin M. Stroebel, Bradley E. Aouizerat, Elena Flowers

**Affiliations:** 1 Department of Physiological Nursing, School of Nursing, 8785 University of California, San Francisco, CA, USA; 2 Keck Graduate Institute, Claremont, California; 3 Department of Obstetrics, Gynecology, and Reproductive Sciences, University of California, San Francisco, CA, USA; 4 Institute for Human Genetics, 8785 University of California, San Francisco, CA, USA; 5 Department of Oral and Maxillofacial Surgery, 5894 New York University, New York, NY, USA; 6 5894 Translational Research Center New York University, New York, NY, USA

**Keywords:** ancestry informative markers, population substructure, whole genome sequencing

## Abstract

**Introduction:** This paper provides an overview of methods for measuring genetic ancestry, specifically focusing on applications for nurses engaged in research. We describe methods and current tools widely implemented by the research community to introduce nurses who conduct research in the landscape of measuring genetic admixture for individuals and genetic substructure for populations. The intended impact of this paper is to enhance awareness and understanding of the importance of measuring genetic ancestry to control for latent confounding in genetic association studies. **Summary of best practices:** Measurement of genetic ancestry can prevent confounding in genetic association studies. **Conclusions:** Nurses approach health from a holistic perspective that includes information about individual, environmental, and social factors. This framework necessitates consideration of individual genetic characteristics and social identity and position. This paper serves as a primer on concepts related to genetic ancestry, including ancestry informative markers, reference populations, and statistical approaches, that nurses engaged in research may incorporate into their study design and implementation.

## Introduction

Whole genome sequencing (WGS) represents a significant technological advancement, enabling the characterization of an individual’s complete set of intrinsic genetic features (Bagger et al., 2024). The most common application of WGS is the evaluation of risk for complex conditions, many of which are the leading causes of morbidity and mortality in the United States (e.g., cardiovascular diseases, diabetes; Murphy et al., 2023). However, another valuable application of WGS technology is the evaluation of geographic ancestry based on an individual’s unique genetic characteristics. This can be achieved by analyzing haplotypes, physically adjacent regions of the genome typically inherited as units during reproduction. Across generations, transmission of these units happens through linkage disequilibrium (Table 1), in which sections of the genome are more likely to be inherited in units than expected by chance (Good, 2022). Mapping these haplotypes provides information about genetic commonalities inherited over generations within specific geographic regions and enables inferences about unmeasured genetic loci that enhance the efficiency of genome sequencing.

**Table 1.** Terms and Definitions

Term	Definition
Ancestry informative markers (AIMs)	Genetic variations (usually SNPs) show substantial differences in allele frequencies between populations from different geographic regions, allowing researchers to infer the ancestral background of individuals
Genetic admixture	The process by which two or more previously isolated and genetically distinct populations begin reproducing together. This results in the creation of new genetic lineages in the population. This often occurs due to migration and resulting mixing of populations
Genetic drift	Random variations during the process of reproduction or other random events that result in a change in the prevalence of a genetic characteristic
Genome wide association study (GWAS)	A study design that explores the genomes of many individuals to identify genetic variations (typically SNPs) that are associated with a particular trait or disease. GWAS help identify risk loci but may be biased if population stratification is not included as a covariate in modeling
Haplotype	A sequence of alleles (i.e., specific loci for a set of DNA variations) that occur on a single strand of DNA that are frequently inherited together, termed linkage disequilibrium. Haplotypes can provide information about ancestry, disease association, and evolutionary history
Linkage disequilibrium (LD)	The non-random association of alleles at different loci in the same chromosomal region in a population. When alleles are in LD, the presence of one allele can predict the presence of another nearby allele. LD patterns vary by population and are important for mapping genetic risk factors
Population substructure	The presence of distinct genetic subgroups withing a larger population, usually due to different ancestry origin or historical patterns of reproduction and migration. If unaccounted for, population substructure can lead to confounding in genetic studies
Recombination	Exchange of genetic information between two homologous regions of the genome. The regions are retained after recombination but swapped between chromosomes, resulting in a new combination of genetic information compared to the parental chromosomes
Selection	The process by which genetic characteristics become more prevalent within a population due to an evolutionary advantage for individuals who carry those traits
Single nucleotide polymorphism (SNP)	Genetic variation of a single nucleotide at a specific chromosomal location
Whole genome sequencing (WGS)	A laboratory test that determines the complete DNA sequence of an individual’s genome. WGS captures nearly all genetic variation, including SNPs, insertions/deletions, and structural variants, making it a powerful tool for personalized medicine and research

Variation in allele frequencies across geographic regions is influenced by genetic mechanisms, including recombination, genetic drift, and natural selection (Table 1). In addition to these processes, geographic isolation can also lead to enrichment of specific variation in populations over time (Chang et al., 2018; Good, 2022; Okazaki et al., 2021). The combination of these phenomena can result in a higher prevalence of some genetic characteristics within geographically distinct populations compared to others. Over the past several centuries, the natural occurrence of these events has been outpaced by human migration, resulting in admixture of persons between previously distant geographic regions (Auton et al., 2015). The result of these migration patterns is mixing of genetic material, which occurs in haplotype segments that become progressively smaller with each generation. By mapping specific haplotypes within the genome, we can characterize geographic ancestral origins, referred to as population substructure. For a specific individual, ancestral characteristics are often expressed as proportions of genetic material inherited from multiple individual ancestral populations that show enrichment of certain genetic characteristics. A well-documented limitation of early genome-wide association studies (GWAS) focused on disease outcomes was the lack of representation of geographically diverse study samples, as many studies were conducted in the United States and Europe (Chang et al., 2018). Genetic variants often differ in frequency across ancestral groups, meaning that some variants are common in one population but are rare or absent in others. As a result, the lack of ancestral diversity in GWAS-based studies can lead to biased or incomplete findings, as genetic risk for a given health outcome will only be captured in the ancestral groups represented in the study sample. This creates a “we don’t know what we don’t know” scenario in which latent confounding can bias results for individuals from populations that were not included in the GWAS samples from which genetic risk factors were identified. When this occurs, individuals may be misclassified as low risk not because they lack risk variants, but because those variants have yet to be identified in their ancestral group(s). An approach to addressing latent confounding by ancestry is using ancestry informative markers (AIMs), which measure geographic ancestral origin based on patterns of linkage disequilibrium (Shriner, 2013). These methods allow researchers to better assess the generalizability of study findings based on whether observed associations between genetic risk factors and health outcomes have been tested in study samples with similar ancestral backgrounds.

**Figure 1.**
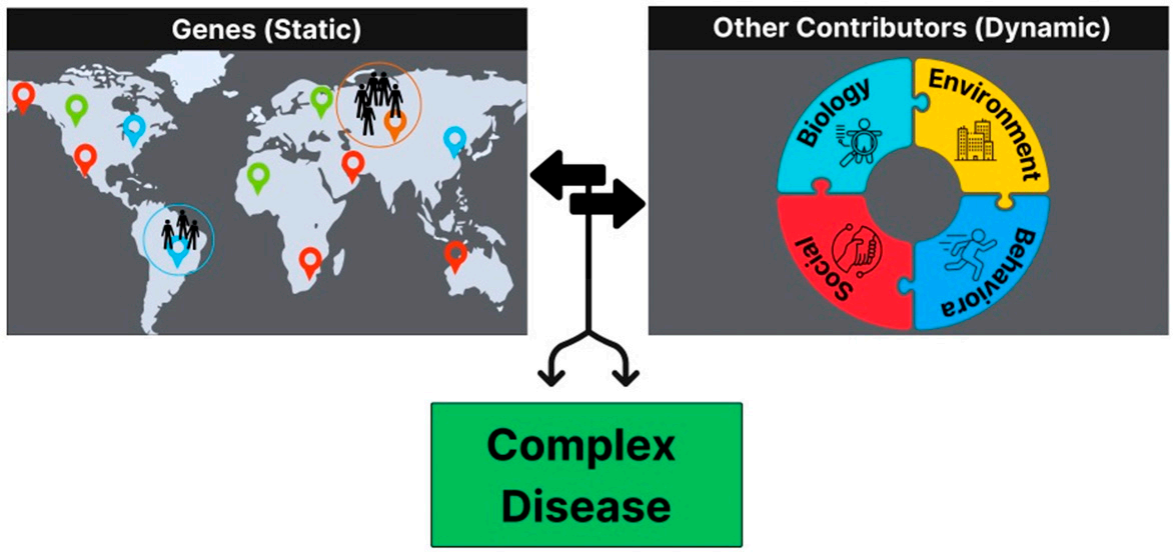
Markers Show Example Geographic Regions From which Populations Originate. Genetic Characteristics are Determined by These Ancestral Regions and Interact With Biological, Social, Environmental, and Behavioral Factors to Determine Risk for Complex Disease. Markers Show Example Geographic Regions From which Populations Originate. Genetic Characteristics are Determined by These Ancestral Regions and Interact With Biological, Social, Environmental, and Behavioral Factors to Determine Risk for Complex Disease

While AIMs are useful for leveraging intrinsic genomic data to assess risk for health outcomes, extrinsic risk factors (e.g., social, environmental) are often also significant contributors (Figure 1). Until recently, health research often conflated the biological construct of ancestry with the social constructs of race and ethnicity. The advent of AIMs and their respective statistical methods has helped address this shortcoming by enabling the quantification of ancestry independent of socially defined constructs. While it is well understood that race and ethnicity are associated with health outcomes, these associations are often a reflection of lived experiences that are shaped by structural and systemic inequities. However, the biological footprint of such experiences has been difficult to quantify because social constructs do not capture the heterogeneity within and across different race and ethnic groups. AIMs present a methodological approach to disentangle biological ancestry from social determinants, allowing for more accurate testing of causal associations between genetic loci and disease outcomes.

There is broad consensus about the importance of measuring ancestral population characteristics in GWAS, which is summarized in a recent report from the National Academy of Sciences that provides a framework for population descriptors in genetics and genomics research (National Academy of Sciences, Engineering, and Medicine, 2023). These practices are now widely being implemented in large-scale research studies that leverage national and international datasets. For example, a recent study that leveraged data from *All of Us Research Program* showed that self-identified race and ethnicity do not precisely align with underlying genetic ancestry. (Gouveia et al., 2025) This is particularly true for individuals who self-identified as Hispanic or Latino who were represented by a wide gradient across principal components of ancestry. This study also validated previous findings that there is greater genetic variation within race and ethnic groups compared to across groups, further supporting the need to accurately define biological ancestral characteristics rather than using race and ethnicity as a proxy estimate for ancestry in order to inform generalizability of study findings and prevent confounding in estimates of the variables that determine causal relationships.

This paper provides an overview of methods for measuring genetic ancestry, with a specific focus on applications for nurses engaged in research. Numerous methods for quantification of percentages of ancestral genetic characteristics and population substructure have been developed, tested, and described in prior review papers focused primarily on computational frameworks and statistical implementation (Enoch et al., 2006; Liu et al., 2013; Shriner, 2013). Given the breadth and complexity of these methods, a comprehensive review of these tools in their entirety is beyond the scope of this paper. Instead, we will describe seminal methods and current “gold standard” tools that are widely implemented by the research community in order to provide nurses who conduct research with an introduction to the landscape of population substructure and implications for design of research studies and eventual clinical implementation. The intended impact of this paper is to enhance awareness and understanding of the concept of genetic admixture among nurses who conduct research, encouraging consideration for integration in future studies that include genetic data (Table 2).

**Table 2.** Evaluation Criteria for Approaches to Measuring Ancestral Genetic Characteristics

Evaluation criteria	Notes
Year developed/published	The contributions of earlier methods are important for understanding the overall trajectory of development of methods for assessing genetic percentages of ancestral genetic characteristics. There may be limitations of some older methods for more admixed populations
Statistical methods/expertise required	Some methods are more “off the shelf” and ready for application by an individual trained in using appropriate statistical software, whereas other methods may offer more opportunities for customizing analysis but require a greater level of expertise in statistical modelling and programming and bioinformatics
Computing environment required	Some analyses will need to be run on an external multi-cluster computing resource; these are available at many academic institutions and from commercial service providers; some may have associated access restrictions and costs. Some methods are designed to be run in specific statistical software (e.g., R, Python) or environments (e.g., Linux)
Populations included	Earlier methods had more limited representation of global population groups and may not be designed to capture the breadth of population substructure now annotated in the 1000 genomes project reference database. Likewise, other methods were developed specifically to assess substructure within distinct ancestral groups (e.g., (Mao et al., 2007; Smith et al., 2004; Tandon et al., 2011; Tian et al., 2006; Wang et al., 2019))
Number of subgroups included	There is variability in the number of ancestral subgroups (K) that can be evaluated between statistical approaches
Genetic data types that can be analyzed	Specific approaches may be appropriate only for some genetic measurement data types, including array-based data, whole genome sequencing, and whole exome sequencing

## Reference Populations

Initial methods for characterization of ancestry were anchored to databases of the genetic characteristics of discrete ancestral groups to serve as a reference for an individual’s measured genetic characteristics. A significant milestone for methods to quantify ancestry was the international HapMap consortium (International HapMap, 2003), which was a global partnership of researchers that defined reference genetic information for populations who originated from Europe (Utah), Asia (Han Chinese, Japanese), and Africa (Yoruban Nigerian). While the initial HapMap reference data presented a significant methodological advance for using genetic data and assessing disease risk, it remained somewhat limited in its global geographic representation. Ultimately, a third phase of HapMap includes 11 subgroups with a greater representation of geographic origins of human populations.

**Figure 2.**
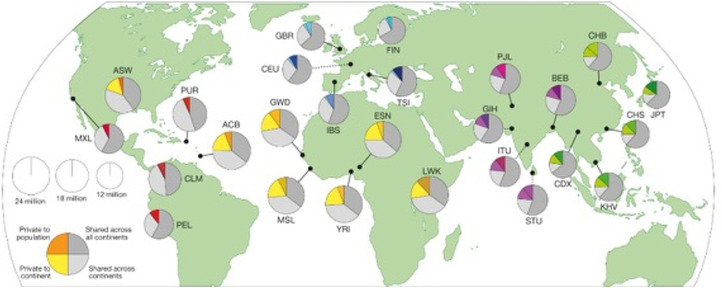
The Area of Each Pie is Proportional to the Number of Variants Within a Population. Pies are Divided Into Four Slices, Representing Variants Private to a Population (Darker Color Unique to Population), Private to a Continental Area (Lighter Color Shared Across Continental Group), Shared Across Continental Areas (Light Grey), and Shared Across all Continents (Dark Grey). Dashed Lines Indicate Populations Sampled Outside of Their Ancestral Continental Region. Adapted From 1000 Genomes Project Consortium, 2015. The Area of Each Pie is Proportional to the Number of Variants Within a Population. Pies are Divided Into Four Slices, Representing Variants Private to a Population (Darker Color Unique to Population), Private to a Continental Area (Lighter Color Shared Across Continental Group), Shared Across Continental Areas (Light Grey), and Shared Across All Continents (Dark Grey). Dashed Lines Indicate Populations Sampled Outside of Their Ancestral Continental Region. ACB – African Caribbean in Barbados; ASW – African Ancestry in Southwest USA; BEB – Bengali in Bangladesh; CDX – Chinese Dai in Xishuangbanna, China; CEU – Utah Residents (CEPH) With Northern and Western European Ancestry; CHB – Han Chinese in Beijing, China; CHS – Southern Han Chinese; CLM – Colombian in Medellin, Colombia; ESN – Esan in Nigeria; FIN – Finnish in Finland; GBR – British in England and Scotland; GIH – Gujarati Indians in Houston, Texas, USA; GWD – Gambian in Western Division – Mandinka; IBS – Iberian Populations in Spain; ITU – Indian Telugu in the UK; JPT – Japanese in Tokyo, Japan; KHV – Kinh in Ho Chi Minh City, Vietnam; LWK – Luhya in Webuye, Kenya; MSL – Mende in Sierra Leone; MXL – Mexican Ancestry in Los Angeles, California, USA; PEL – Peruvian in Lima, Peru; PJL – Punjabi in Lahore, Pakistan; PUR – Puerto Rican in Puerto Rico

Building on the HapMap project, the 1000 Genomes Project (1 kg) (Genomes Project et al., 2015) contains 26 reference populations from 2,054 (Figure 2) and is typically used as the current gold standard for mapping global population substructure. These data are widely available to researchers with appropriate training and resources to ensure data security through controlled access agreements. The overall impact of these tools is that individual ancestry can be measured and operationalized as a quantitative variable for statistical modelling and evaluation of genetic causal mechanisms for health outcomes.

## Methods for Analysis of Ancestral Genetic Characteristics

There are many criteria by which evaluation of potential statistical approaches to define genetic admixture might be evaluated (Table 2). Decisions about which tool to select will depend on the population of interest, research question, expertise of the research team, access to resources, and a tradeoff between generalizability and specificity of the findings. Several tools have been developed that specifically look at population substructure within distinct ancestral groups, including Latin/Hispanic, Asian, and African. (Ding et al., 2000; Mao et al., 2007; Mörseburg et al., 2016; Smith et al., 2004; Tandon et al., 2011; Tian et al., 2006; Wang et al., 2019) This is somewhat conflated with the constructs of race and ethnicity given that individuals in these studies are determined to be eligible based on self-report, and membership in an ancestral group can’t be defined until the participant’s genetic data have been measured. This differentiation between individual or social-level identification of race and ethnicity from genetic ancestry is one of the essential strengths of using AIMs to control for population substructure and prevent confounding by ancestry. Including population substructure as a covariate creates the opportunity to look at the potential impacts of social determinants (i.e., race and racism) on health outcomes, distinct from genetic characteristics associated with geographic ancestry.

**Figure 3.**
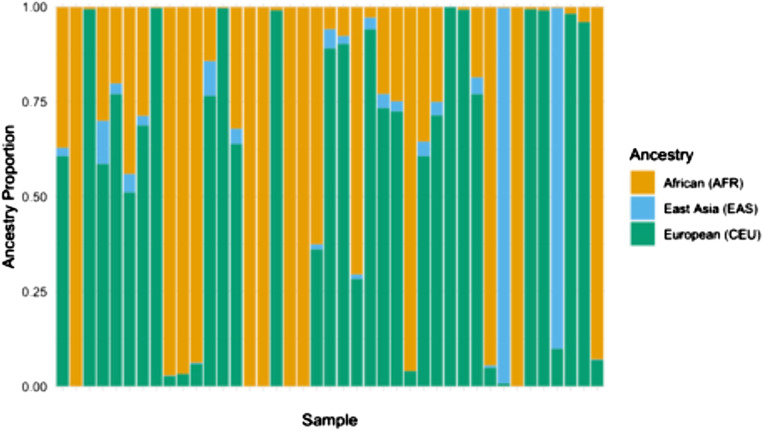
Estimation of Ancestral Proportion in Clinical Trial Participants. Stacked Bars Show Estimated Ancestry Proportions for Each Individual (n = 41). ADMIXTURE Analysis was Performed Using Genotype Data Merged With HapMap 3 References Samples, With the Number of Ancestral Populations (K) Set to 3. Each bar Represents a Single Individual, and the Color Segments Within Each bar Correspond to the Proportion of Ancestry in Three Inferred Ancestry Clusters: African (AFR, Orange), East Asian (EAS, Light Blue) and European (CEU, Green). Estimation of Ancestral Proportion in Clinical Trial Participants. Stacked Show Estimated Ancestry Proportions for Each Individual (*n* = 41). ADMIXTURE Analysis was Performed Using Genotype Data Merged With HapMap 3 References Samples, With the Number of Ancestral Populations (K) Set to 3. Each bar Represents a Single Individual, and the Color Segments Within Each bar Correspond to the Proportion of Ancestry in Three Inferred Ancestry Clusters: African (AFR, Orange), East Asian (EAS, Light Blue) and European (CEU, Green)

STRUCTURE was one of the first published methods to analyze percentages of ancestral genetic characteristics. This approach applies models based on clustering probability and prior admixture distributions to determine the likelihood that a given allele is present at a given locus. (Pritchard et al., 2000) This method allows for deriving K number of ancestral groups, which can be pre-specified or unknown. The result is K groups with shared genetic characteristics, which can be evaluated within an independent sample or referenced to annotated ancestral groups to determine the presence and proportion of ancestry for an individual (Figure 3). The data can also be aggregated to show a summation of ancestral characteristics for a subgroup or study sample. Building on STRUCTURE, a current widely used approach is ADMIXTURE. (Alexander et al., 2009) Mathematical modifications integrated into ADMIXTURE include cross-validation to estimate the value of K rather than calculating model estimates for each value of K, which results in determining the number of K with the best predictive value and, importantly, notably faster computational speeds. ADMIXTURE has nearly 4,000 citations, evidence of its wide uptake and broad utilization. Both STRUCTURE and ADMIXTURE are publicly available tools (Alexander et al., 2009; Pritchard et al., 2000). Processing times vary depending on the number of genetic loci and number of individuals included in a dataset.

Another widely used approach for estimating genetic ancestry is Principal Component Analysis (PCA). PCA is a statistical technique that can reduce the dimensionality if genomic data by summarizing variation across the genome into principal components. These principal components represent an axis of genetic variation and often align with geographical ancestry allowing for researchers to detect population substructure without predefined labels. PCA is frequently used in GWAS to correct for ancestry related confounding and is considered a computationally efficient method for identifying major axes of genetic variation (Privé et al., 2020). Many commonly used statistical software programs have the capability of performing PCA using genomic datasets.

**Figure 4.**
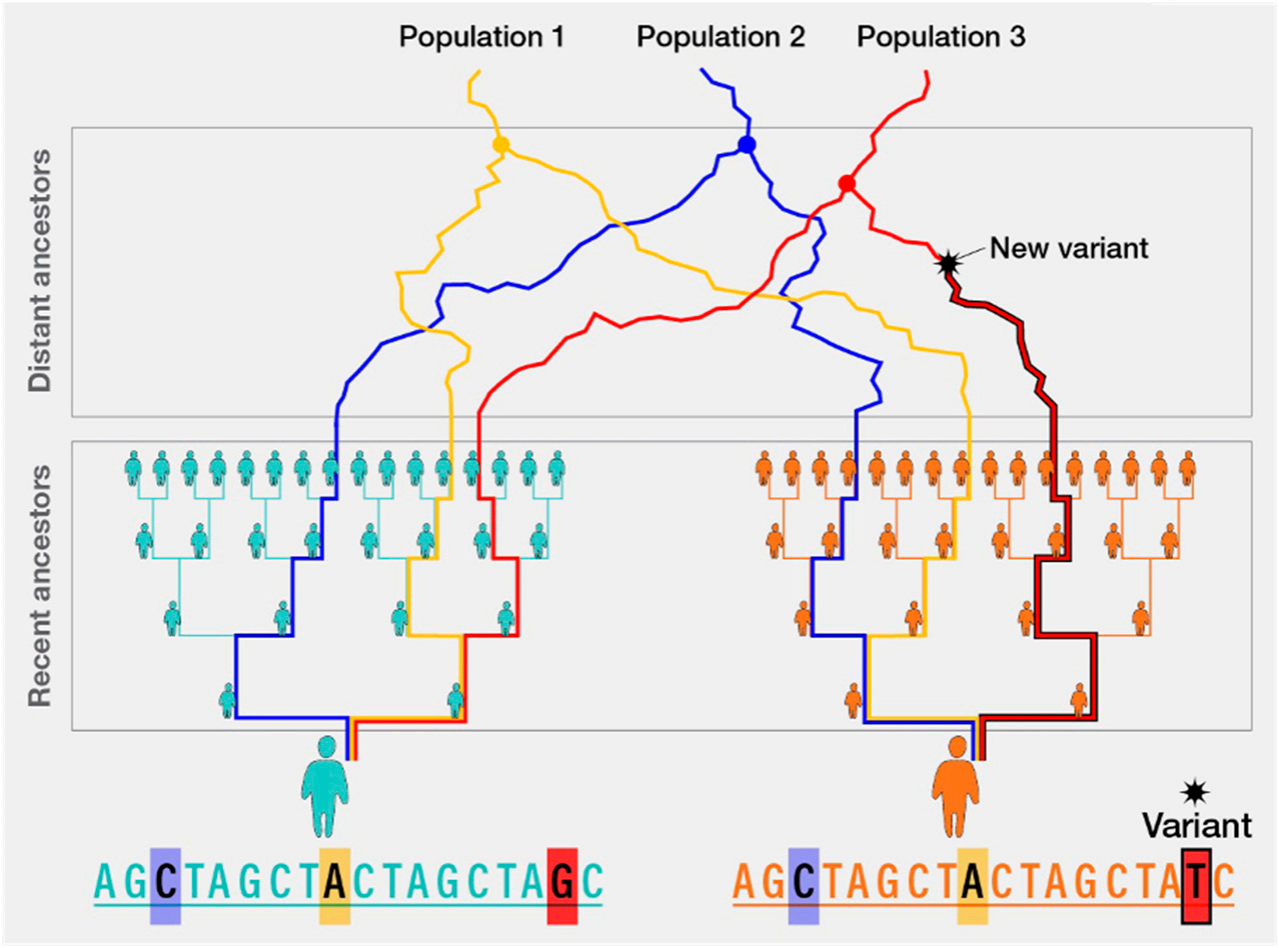
Broad Global Origin Populations are Shown as Distinct Ancestors. New Variants can be the Result of Recombination, Genetic Drift, and Selection. Recent Ancestors Show More Recent and More Rapid Admixture in Specific Populations (e.g., Africans in America, Latin/Hispanic With Mapping of Distant and Recent Ancestral Groups Within Specific Regions of the Genome. Adapted From https://www.genome.gov/genetics-glossary/Local-Ancestry Broad Global Orgin Populations are Shown as Distinct Ancestors. New Variants can be the Result of Recombination, Genetic Drift, and Selection. Recent Ancestors Show More Recent and More Rapid Admixture in Specific Populations (e.g. Africans in America, Latin/Hispanic With Mapping of Distant and Recent Ancestral Groups Within Specific Regions of the Genome

Both STRUCTURE and ADMIXTURE provide methods for understanding ancestry at a genome-wide level, providing estimates of global ancestral composition across an individual genome. In contrast, other statistical tools are focused on estimating ancestry on a local scale, meaning at a specific position (i.e., a large segment of DNA form a small region of a chromosome) within the genome. This allows for assessment of the ancestral group composition for a particular genetic region of interest and determination of the ancestral origin of each allele at a given locus. These methods were developed to more accurately characterize admixed populations including individuals who are typically categorized as Latin/Hispanic and African American/Black -- groups that may have Native American, African, and European ancestral origins. (Baran et al., 2012; Zakharia et al., 2009) These methods allow a more rigorous assessment of the likelihood that a given risk allele occurs in an individual, considering normal biological processes like recombination, genetic drift, and selection (Figure 4; Goli et al., 2024). The most common methods applied for inference of local ancestry is Local Ancestry in Mixed Populations (LAMP; Baran et al., 2012), which assesses individual SNPs for their reference population ancestral group prevalence and summarizes the most probable ancestral group across multiple SNPs within a specific region of the genome. Like the global ancestry tools described above, LAMP is also available in the public domain (Baran et al., 2012).

## Discussion of Implications for Nurses and Researchers

Historically, health sciences research has often conflated constructs of ancestry versus race and ethnicity. However, scientific discoveries over the past several decades have provided an opportunity to differentiate these into distinct domains of risk with improved ability to measure the distinct contributions of genetic risk factors to health outcomes based on ancestral composition. By applying well-tested scientific theories and leveraging the rigorous methods described above, nurses who are engaged in research are positioned to conduct studies that have the potential to significantly improve our understanding of latent causal relationships that are masked when the constructs of ancestry and race and ethnicity are conflated. Defining population substructure with GWAs can decrease the likelihood of both false positive and false negative conclusions, inform interpretation of validation of research findings, about provide more accurate information about generalizability of findings across study populations. This has a direct impact on potential clinical implementation of genetic information to reach appropriate patient populations in order to optimize treatments and minimize potential harms.

Both genetic characteristics (ancestry) and race and ethnicity may be instrumental variables that represent more complex and nuanced exposures that impact health outcomes. This is evidenced by the principle of linkage disequilibrium in the context of genetic variables in which a relatively small number of genetic loci act as instrumental variables that represent the genetic characteristics of entire region of the genome. In terms of social exposures, race and ethnicity have been used as instrumental variables to estimate social experiences and opportunities in the absence of being able to measure these exposures at a more accurate and granular level. The ultimate goal should be to move beyond race and ethnicity through development of methods and tools that accurately capture the precise social exposures (e.g., microaggressions, perceived stress) that directly impact health outcomes on an individual rather than broadly aggregated but poorly refined group level.

One important caveat to the concept of reference populations is that historical human actions have driven the rate and directions of migration patterns. In some cases, groups of individuals have been relocated forcefully with corresponding cultural and social assimilation that may impact social identity, distinct from ancestry. These events have contributed to what is inherently the definition of genetic admixture and underlie the rationale for accurate assessment of the likelihood that genetic loci associated with disease outcomes will be present in an individual or group of individuals with shared ancestry. However, the naming of ancestral groups by global regions may erroneously include or exclude groups of individuals who were forcefully relocated. This current limitation is an important consideration for continued development of methods to control for the likelihood of prevalence of relevant genetic loci when evaluating overall genetic risk for health outcomes. For example, reference databases could subvert the issue of forced migration and related cultural assimilation by using a nomenclature structure that is based on genetic structure rather than global geographic region. This can be accomplished through agnostic statistical approaches that derive K groups based on shared genetic characteristics within a sample rather than referenced to geographically anchored prespecified groups. Alternatively, a repository of reference groups could be defined and named based on an arbitrary naming schema or one that uses genetic structural characteristics like haplotypes.

Nurses have historically approached health from a holistic perspective that includes information about the individual, the immediate and distal environments, and higher-level social factors. This framework necessitates consideration of individual-level factors including genetic characteristics and social identity and position. Increasingly, genetic information is being collected in research studies, both for immediate application to research questions and in biobanks for future applications. This facilitates collection of information to define genetic ancestry and population substructure through AIMs. The methods for generating these ancestry variables have been rigorously developed and tested and are highly accessible in the public domain. Current best practices include the 1 kg reference database and the ADMIXTURE and LAMP modeling approaches, although methods will inevitably continue to evolve over time.

## Conclusions

When considering the etiology of complex diseases, it is important to apply rigorous data collection approaches for to measure complex variables that may be instrumental variables for multi-faceted exposures. The human genome project was an international collaborative that provided significant technological advancements, including the possibility for quantification of geographic ancestry of an individual, distinct from their self-reported or assigned race and ethnicity, through measurement of population substructure based on the principle of linkage disequilibrium. More recent methods expand on these discoveries by allowing for determination of ancestral origin of just a specific region of the genome, termed local ancestry. Genetic data are increasingly available from research studies and can be used in causal models to evaluate health outcomes and control for genetic ancestry and population substructure, distinct from social constructs that also impact health outcomes. This approach is precisely aligned with frameworks utilized by nurses who do research that include person, environment, and social-level contributors to health outcomes. This paper serves as a primer on applying these methods, including ancestry informative markers, reference populations, and statistical approaches, that nurses engaged in research may incorporate into their study design and implementation.

## Data Availability

No data are presented in this paper.*
